# Nano-Precision Processing of NiP Coating by Magnetorheological Finishing

**DOI:** 10.3390/nano13142118

**Published:** 2023-07-20

**Authors:** Chao Xu, Xiaoqiang Peng, Hao Hu, Junfeng Liu, Huang Li, Tiancong Luo, Tao Lai

**Affiliations:** 1 Laboratory of Science and Technology on Integrated Logistics Support, College of Intelligence Science and Technology, National University of Defense Technology, Changsha 410073, China; wjsxcr@126.com (C.X.); pxq2000@vip.sina.com (X.P.); tiny_hh@139.com (H.H.); ljf20090702122@163.com (J.L.); li_huang6002@163.com (H.L.); 2 Beijing Zhenxing Institute of Metrology and Measurement, Beijing 100074, China; tcluo@163.com

**Keywords:** NiP coating, magnetorheological finishing, single-point diamond turning, process parameters, surface roughness

## Abstract

NiP coating has excellent physicochemical properties and is one of the best materials for coating optical components. When processing NiP coatings on optical components, single-point diamond turning (SPDT) is generally adopted as the first process. However, SPDT turning produces periodic turning patterns on the workpiece, which impacts the optical performance of the component. Magnetorheological finishing (MRF) is a deterministic sub-aperture polishing process based on computer-controlled optical surface forming that can correct surface shape errors and improve the surface quality of workpieces. This paper analyzes the characteristics of NiP coating and develops a magnetorheological fluid specifically for the processing of NiP coating. Based on the basic Preston principle, a material removal model for the MRF polishing of NiP coating was established, and the MRF manufacturing process was optimized by orthogonal tests. The optimized MRF polishing process quickly removes the SPDT turning tool pattern from the NiP coating surface and corrects surface profile errors. At the same time, the surface quality of the NiP coating has also been improved, with the surface roughness increasing from Ra 2.054 nm for SPDT turning to Ra 0.705 nm.

## 1. Introduction

High-precision optical mirrors are widely used and in demand in fields such as Earth observation [1], space telescopes [2], laser fusion [3], and the manufacture of large-scale integrated circuits [4,5], where the characteristics of high surface shape accuracy and high surface quality requirements place higher demands on modern optical processing technology. Compared to reflectors made of brittle materials such as fused silica and microcrystalline glass, metallic mirrors represented by aluminum alloys have unique advantages [6,7]. The support structure in the optical system is also metal, and the support element and the reflector can be made of the same metal, which can avoid thermal stresses caused by different coefficients of thermal expansion between the support structure and the reflector material [8,9]. At the same time, metal mirrors can be used for broad-spectrum applications because they cover a wide spectral range [10]. In addition, metallic materials are easy to shape and far less expensive to process than reflectors made of brittle materials such as fused silica and microcrystalline glass [11]. Therefore, metal mirrors have important application value.

Visible and shorter wavelength spectroscopy applications require optical components with high surface quality. Hence, electroless nickel plating is often applied to improve the processability of the mirrors [12]. NiP coating is typically thickened with tens to a hundred microns and is an amorphous coating with high phosphorus content. As a consequence, NiP coating has excellent machinability [13]. After covering the NiP coating on the reflector substrate with an electroless nickel plating method, the first process is the SPDT machining of the NiP coating [14,15]. SPDT is an excellent machining method with high efficiency, low cost, and ease of forming complex surfaces. Therefore, SPDT turning is the most commonly used method for machining metallic optical components [16,17,18]. However, SPDT turning leaves periodic turning tool patterns on the surface of the workpiece, which can increase light scatter and degrade the system’s image quality [19,20]. Thus, mirrors machined with SPDT are typically used in the infrared spectral region and must polish the mirror for applications in the visible and shorter wavelength spectral range [21].

Optical polishing of the NiP coating surface after SPDT turning can improve surface quality while removing residual tool marks. For example, Namba et al. [22] of Chubu University, Japan, investigated the ultra-precision fabrication of electroless nickel plating aspheric forming molds for hard X-ray telescope mirrors, using an SPDT turning and floating polishing process to obtain a surface roughness of RMS 0.228 nm. Tinsley [23] reached a surface roughness better than RMS 0.1 nm using SPDT ultra-precision turning followed by chemical mechanical polishing using specific slurry and processing parameters on off-axis aspheric mirrors coated with NiP. While float polishing and chemical mechanical polishing of the NiP coating resulted in good surface roughness, the low polishing efficiency resulted in a longer time spent on the whole process.

As an advanced optical manufacturing technology, MRF differs from the pressure-driven material removal of conventional polishing. MRF has a unique shear removal mechanism that enables material removal at the micron or even nanometer scale, thus can obtain ultra-smooth surfaces with nano-precision [24,25,26,27,28]. During the MRF process, the magnetorheological fluid cycled into the machining area, and the internal magnetorheological fluid and abrasive particles are continuously renewed circularly, taking away the heat and material debris generated by the machining and maintaining the stability of the machining area [29]. The low normal pressure of MRF improves the surface quality of the workpiece and removes residual stresses when correcting surface shape errors [30]. MRF does not require high support methods of workpiece or ambient temperatures, and error-influencing factors that are difficult to control in conventional polishing, such as the polishing tool’s deformation and wear, have less impact on MRF [31]. MRF enables efficient and high-precision machining of optical mirrors and is widely used in optical machining. Therefore, MRF can be used to remove turning tool patterns from NiP-coated surfaces [32,33].

In this paper, we investigate the MRF process for machining NiP coating that can rapidly remove SPDT turning patterns while correcting surface shape errors. In Section 2, we analyze the material properties of the NiP coating and develop a special magnetorheological fluid. A material removal model for MRF of NiP coating was established based on the basic Preston principle. In Section 3, the effects of magnetic field strength, polishing wheel rotation speed, press-in depth of ribbon, and polishing fluid flow rate on material removal are investigated by orthogonal tests and used to optimize the MRF process. In Section 4, we verify the optimized process, eliminate the SPDT turning tool marks on the surface of NiP coating, and correct the surface shape errors by MRF. At the same time, MRF improved the surface quality of the workpiece and reached a roughness of better than Ra 1 nm. MRF enables efficient and high-precision machining of NiP coating.

## 2. Materials and Equipment

### 2.1. The Characteristics of NiP Coating

The NiP coating in this paper was deposited uniformly onto the aluminum alloy substrate (Al 6061T6, Al 97.8 wt%, Mg 0.96 wt%, Si 0.59 wt%, Fe 0.30 wt%, Cu 0.21 wt%, Mn 0.09 wt%, Zn 0.02 wt%, else 0.03 wt%) by electroless nickel plating and is a high-phosphorus coating with a P content of about 10% wt. Electroless nickel plating was carried out in a plating solution in which a chemical reaction took place, with a pH value of 6.0, a composition of NiSO_4_-6H_2_O, NaH_2_PO_2_-H_2_O, and CH_3_COONa-3H_2_O as the main components of the plating solution, a plating temperature of 90 °C, and a plating speed of 10 μm/h.

**Figure 1:**
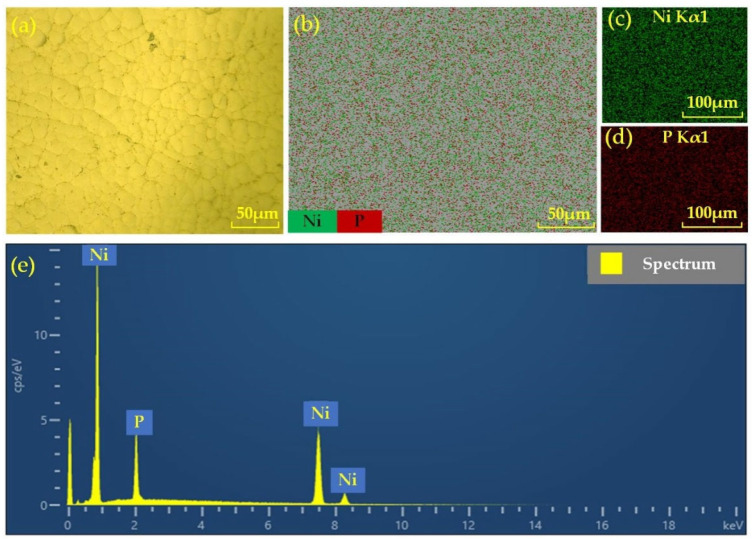
Test results of NiP coating surface: (**a**) surface morphology of NiP coating; (**b**) SEM mapping for the selected area; (**c**) mapping for Ni; (**d**) mapping for P; (**e**) EDS spectrum analysis.

Figure 1a shows the surface of the NiP coating without any treatment observed under a digital microscope (Keyence VHX-600E, Osaka, Japan), and it can be noticed that the coated presents the typical nodular features of cauliflower-like structures, which are Ni-P nodules formed on the coating catalyzed by hydrogen atoms [34]. Figure 1a shows that the entire surface is homogeneous and dense, without any defects such as porosity or inclusions. Figure 1b–e shows plots of observations of the NiP coating using a scanning electron microscope (TESCAN MIRA LMS, Czech Republic). From Figure 1b–e, it can be found that the coating is mainly composed of Ni and P. The distribution of the two elements is quite uniform, and the surface has few impurities and good processability.

**Figure 2:**
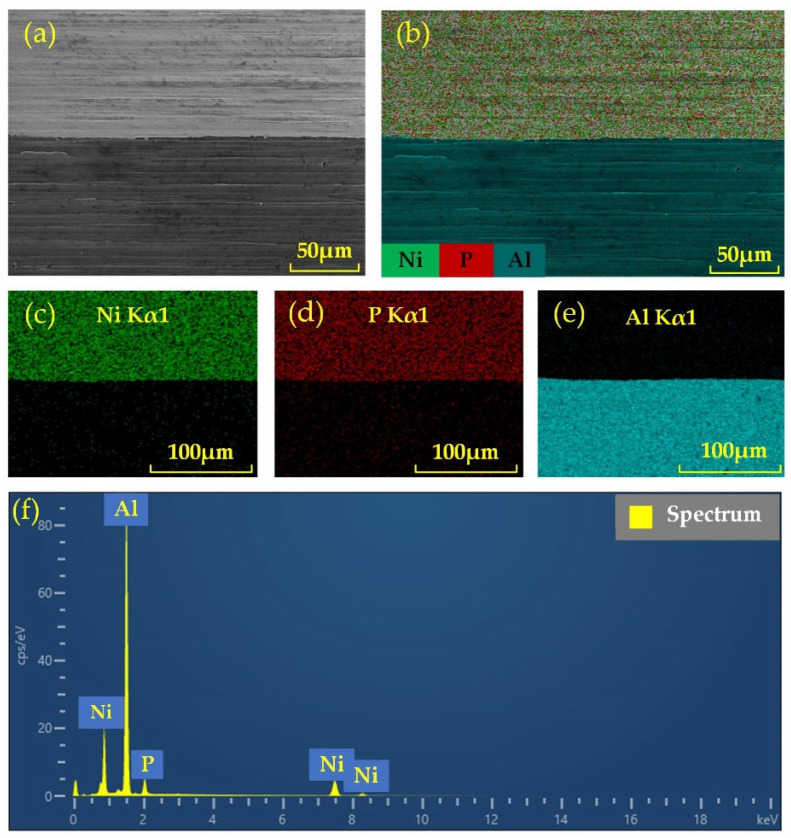
Test results of NiP coating cross-section: (**a**) SEM image; (**b**) mapping for the selected area; (**c**) mapping for Ni; (**d**) mapping for P; (**e**) mapping for Al; (**f**) EDS spectrum analysis.

Figure 2a–f shows the cross-section of the NiP coating observed under the scanning electron microscope (TESCAN MIRA LMS, Brno, Czech Republic). To observe the NiP coating cross-section, we used wire-cutting technology to cut a test sample with a diameter of 10 mm and a thickness of 5 mm according to the size requirements of the test sample for SEM. Then, the sample was cleaned using an ultrasonic cleaner, dried in a drying oven after cleaning, and sealed and stored to avoid impurities from interfering with the test.

From Figure 2a, it can be found that the thickness of the coating is about 100 μm. It is important to note that the coating must not be too thin, as this can lead to defects such as holes in the machined surface when ultra-precision turning and polishing processes. From Figure 2a, it can be found that the bond between the coating and the aluminum alloy substrate is very well, there are no gaps, cracks, or other defects at the bonding surface, and the coat is uniform, dense, and has strong adhesion. From Figure 2b–f, it can be found that the elements in the coating are mainly Ni and P. The elements in the substrate are mainly Al elements, and Ni and P are uniformly distributed in the layer.

**Figure 3:**
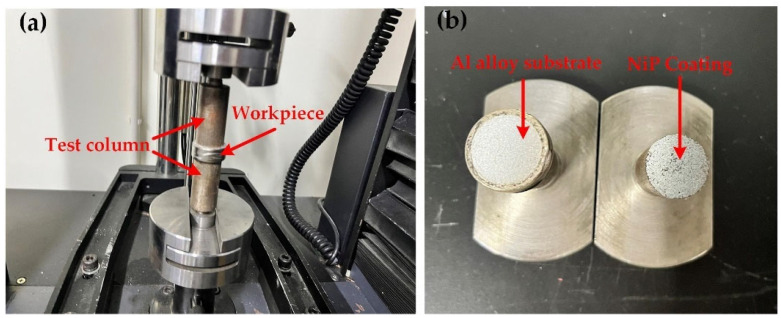
Adhesion test of NiP Coating: (**a**) under testing; (**b**) end of testing.

The bonding force of NiP coating was tested according to Chinese standard GB/T 5210-2006. The testing equipment was an electronic universal material testing machine (UTM6104, Shenzhen Sanshi Zongheng Technology Co., Ltd., Shenzhen, China). During the test, the workpiece was bonded directly to the test column of the tester with adhesive, and after the adhesive was cured, the bonded test column was applied to the tensile tester, as shown in Figure 3a. After a controlled tensile test, the tensile force required to break the adhesion between the coating and the substrate is measured, as shown in Figure 3b for the separated coating and substrate. The following formula was used to calculate the breaking strength of the test combination:

*σ* = *F*/*A* (1)

where *σ* is the breaking strength in MPa, *F* is the breaking force in Newton (N), and *A* is the area of the test column in square millimeters (mm^2^). After testing and calculation, the destructive strength of the coating and the substrate is 18.34 MPa.

From the test results of the surface and cross-section of the NiP coating in Figure 1 and Figure 2, we could find that the chemically plated NiP coating is uniform and dense, and no defects such as cracks and gaps exist on the surface. Meanwhile, through the adhesion test, the breaking strength of the coating and the substrate is as high as 18.34 MPa, and the adhesion between the layer and the substrate material is strong and well-bonded. Therefore, the NiP coating has excellent material properties.

### 2.2. Processing Equipment and Polishing Fluid of MRF

**Figure 4:**
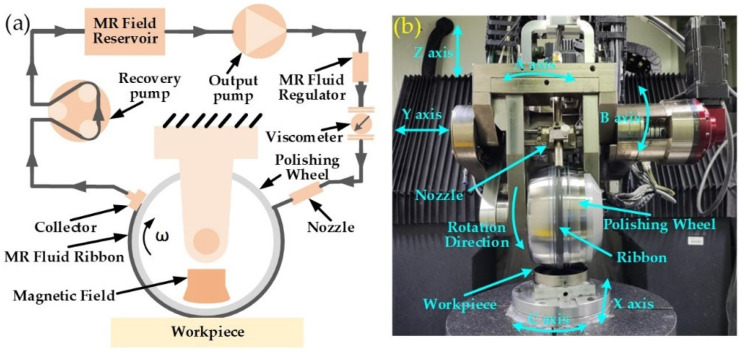
Schematic and device of MRF: (**a**) schematic of the MRF; (**b**) machine tool of MRF.

Figure 4a shows that the MRF equipment mainly consists of polishing wheels, magnets, magnetorheological fluid circulation, and a stability control system. The magnetorheological fluid circulation and stability control system is the key part of the MRF device, which consists of a recycler, a recycling pump, a reservoir, an output pump, a flow meter, a viscometer, and a nozzle. During processing, the magnetorheological fluid is output from the reservoir through the output pump, controlled by the flow meter and viscometer for stability, and then sprayed onto the polishing wheel through the nozzle. The magnetorheological fluid is converted into a solid in milliseconds by the gradient magnetic field, and the workpiece is processed at the bottom of the polishing wheel. After machining is complete, the magnetorheological fluid is recycled to a reservoir via a recycler and recycling pump, completing the cycle. Due to the rheological controllability of MRF, the magnitude of the yield stress in the solid phase state of the magnetorheological fluid can be adjusted by controlling the strength of the magnetic field, which facilitates the generation of tiny cutting effects and enables material removal processing at the micron or even nano level.

As shown in Figure 4b, the MRF machine (KDUPF650-7) was developed by our group. The machine has three linear axes, X, Y, and Z, and three rotation axes, A, B, and C. It is capable of machining flat, spherical, aspherical, and free-form components. The magnetorheological fluid ribbon moves over the surface of the workpiece in a specific path and speed under computer control. By controlling the residence time in each area, the amount of surface material removed can be precisely controlled to correct surface errors and improve accuracy. The optimum range of process parameters for MRF is shown in Table 1.

During the MRF process, the chemical reaction between the magnetorheological fluid and the surface of the workpiece and the mechanical removal of the workpiece by the polishing abrasive occur simultaneously. Therefore, the magnetorheological fluid is one of the crucial factors in achieving high-precision MRF polishing. In this study, a magnetorheological fluid was configured especially for the processing of NiP coating. The primary materials include carbonyl iron powder, silicon carbide polishing abrasive, complexing agent, pH adjuster, and deionized water. The polishing was carried out using a raster scanning path with a step size of 1 mm and a feed rate of 100 mm/min.

Measurement of the face shape error of the workpiece using a laser interferometer (Zygo Verifire, Zygo Corporation, Connecticut, USA). The roughness of the workpiece was measured using a white-light interferometer (Zygo NewView 700S, Zygo Corporation, Connecticut, USA). Both devices are based on the optical interferometry principle. The laser interferometer has an aperture of 6 inches, CCD pixels of 1000 × 1000, wavefront repeatability of <RMS 2 nm, and measurement repeatability of <RMS 0.05 nm. The white-light interferometer has a field of view of 0.07~9.3 mm, a longitudinal resolution of <0.1 nm, and measurement repeatability of <RMS 0.01 nm.

## 3. Analysis of Removal Characteristics of MRF

### 3.1. Material Removal Model of MRF

The theoretical basis for MRF processes is the Preston equation [35]:

∆*H* = *K P V* (2)

where ∆*H* is the amount of material removed per unit of time; *K* is the Preston constant, which is related to factors such as workpiece material, abrasive, and work area temperature; *P* is the positive pressure between the optical part and the polishing tool; and *V* is the relative velocity of the workpiece and the polishing tool.

The factors influencing MRF polishing are complex, according to the findings of researchers at the University of Rochester [31]. The main factors influencing the MRF removal function are workpiece material, magnetorheological fluid, and process parameters. The magnetorheological fluid affects the mechanical properties and chemical stability of the material surface, while the process parameters affect the magnitude and distribution of the magnetic field, pressure, and shear stress in the polished area. Due to the unique material removal mechanism of the MRF, the effect of each factor on the removal function is finally unified into an effective depth of indentation of the abrasive particles.

The forces exerted on the surface of the abrasive grain during MRF consist of gravity, magnetization pressure, and fluid dynamic pressure, where gravity is negligible [36,37].

*P* = *P_m_* + *P_d_* (3)

where *P_m_* is the magnetization pressure generated by the gradient magnetic field, and *P_d_* is the fluid dynamic pressure generated by the flow of the polishing fluid.

(4) $$P_{m}=3\varphi \mu_{0}\mu_{f}\frac{\mu_{P}-\mu_{f}}{\mu_{P}+2\mu_{f}}\int_{0}^{H}BdB$$

where *φ* is the volume ratio of carbonyl iron powder in the polishing solution, *μ*_0_ is the vacuum permeability, *μ_f_* is the magnetic permeability of the magnetorheological fluid, *μ_p_* is the magnetic permeability of carbonyl iron powder in the polishing solution, and *B* is the magnetic field strength.

(5) $$P_{d}=\frac{-2\eta_{0}Ux}{h^{2}}$$

where *η*_0_ is the viscosity of the magnetorheological fluid, *U* is the tangential speed of the polishing wheel, and *h* is the height between the workpiece and the polishing wheel along the *x*-direction.

(6) $$h=h_{0}+r-\sqrt{r^{2}-x^{2}}\approx h_{0}+\frac{x^{2}}{2r}$$

where *h*_0_ is the height of the workpiece to the polishing wheel at *x* = 0, and *r* is the radius of the workpiece.

The gap between the workpiece and the polishing wheel is much smaller than the radius of the polishing wheel. Therefore, we can use the surface speed of the polishing wheel instead of the relative velocity.

(7) V=ω×R=2πn×R

where *ω* is the angular speed of the polishing wheel, *n* is the speed of the polishing wheel, and *R* is the radius of the polishing wheel. Therefore:

(8) $$\Delta H=2K\pi nR\left[\frac{-2\eta_{0}Ux}{h^{2}}+3\varphi \mu_{0}\mu_{f}\frac{\mu_{P}-\mu_{f}}{\mu_{P}+2\mu_{f}}\int_{0}^{H}BdB\right]$$

The material removal mechanism of MRF is mainly plastic removal. The load and depth of indentation of a single abrasive grain largely determine the material removal efficiency and surface quality. The following is a theoretical calculation of the force and depth of indentation on a single abrasive grain.

**Figure 5:**
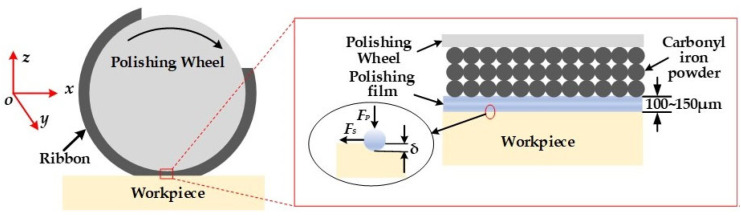
Schematic of the interaction between abrasive particles and component surfaces.

As in Figure 5, the positive pressure on an abrasive with an equivalent diameter of *r*_1_ is identical to the product of the projected abrasive area on the workpiece’s surface and the polishing pressure.

(9) $$F_{P}=\frac{1}{4}P\pi r_{1}{}^{2}$$

where *r*_1_ is the equivalent diameter of the polished abrasive.

The force on the abrasive grain was defined by the size of the abrasive grain as well as the contact pressure between the polishing die and the workpiece. During polishing processes, the contact between the abrasive grain and the workpiece can be considered as a sliding impression of a half-space by a rigid indenter, so the contact between the abrasive grain and the polishing die can be considered as a quasi-static impression of a half-space by a rigid indenter. Based on the state of force on the abrasive grain and the definition of Vickers hardness, the following expression holds [38]:

(10) $$F_{P}=\frac{1}{2}H\pi r_{2}\delta$$

where *H* is the Vickers hardness of the workpiece in the magnetorheological fluid environment, *r*_2_ is the diameter of the edge circle when the abrasive grain is in contact with the workpiece, and *δ* is the depth of the abrasive grain pressed into the workpiece. The depth of the abrasive grain pressed into the workpiece can be obtained by combining Equations (9) and (10) as follows:

(11) $$\delta =\frac{Pr_{1}{}^{2}}{2Hr_{2}{}^{2}}$$

(12) $$\delta =\frac{r_{1}{}^{2}}{2Hr_{2}{}^{2}}\left[\frac{-2\eta_{0}Ux}{h^{2}}+3\phi \mu_{0}\mu_{f}\frac{\mu_{P}-\mu_{f}}{\mu_{P}+2\mu_{f}}\int_{0}^{H}BdB\right]$$

Analysis of Equation (12) shows that for a defined position in the MRF area, the fluid dynamic pressure, the hardness of the workpiece, and the equivalent diameter of the abrasive grain *r*_1_ are generally fixed values. However, the diameter of the edge circle *r*_2_ when the abrasive grain is in contact with the workpiece changes continuously with the contact angle and contact state of the abrasive grain, which leads to changes in the depth of the abrasive grain pressed into the workpiece.

### 3.2. Evaluation of Removal Function

**Figure 6:**
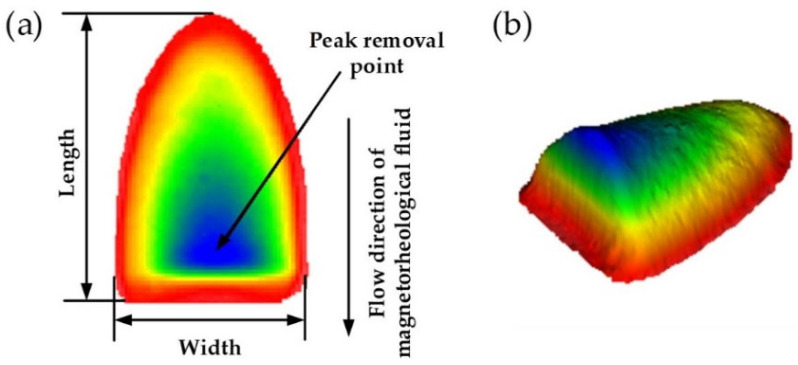
Removal function of MRF: (**a**) 2D diagram; (**b**) 3D diagram.

A typical MRF removal function is shown in Figure 6, whose main evaluation criteria include geometry, removal rate, and surface roughness. The geometry mainly consists of the length and width of the removing function. Removal rates include peak removal rate (PRR, μm/min) and volume removal rate (VRR, mm^3^/min). The peak removal rate is defined as the maximum amount of material removed per unit of time at the peak removal point, which is the peak point in the removal function’s efficiency profile. The volumetric removal rate is the volume of material removed by the removal function per unit of time.

## 4. Experiment

### 4.1. Experimental Setup

Material, magnetorheological fluid, and process parameters can influence the quality of NiP coating through MRF polishing. The main process parameters are the polishing wheel speed, the polishing fluid flow rate, the magnetic field, and the ribbon press-in depth, which can impact the polishing efficiency and surface quality of the NiP coating. We investigated the influence of these process parameters on the removal function to better control the individual process parameters according to requirements during the actual process. For each of the four process parameters—wheel rotational speed, polishing fluid flow, magnetic field, and ribbon indentation depth—three levels of MRF process equipment and actual working conditions would require 81 (3^4^) trials to achieve a comparison of each factor individually [36].

The number of trials is so large that it is difficult to study each factor, so this experiment uses a scientific experimental method—the orthogonal test method. The orthogonal experimental method is a method that uses statistical views, applies the principle of orthogonality, and uses orthogonal tables to arrange the characteristics of equilibrium matching [24,36]. The orthogonal test method can reduce the number of tests, and the processing of test results is more scientific. As shown in Table 2, the four process parameters of polishing wheel speed, fluid flow rate, magnetic field, and ribbon press-in depth were used to form a four-factor, three-level orthogonal test at three levels.

### 4.2. Orthogonal Test of Process Parameters

**Figure 7:**
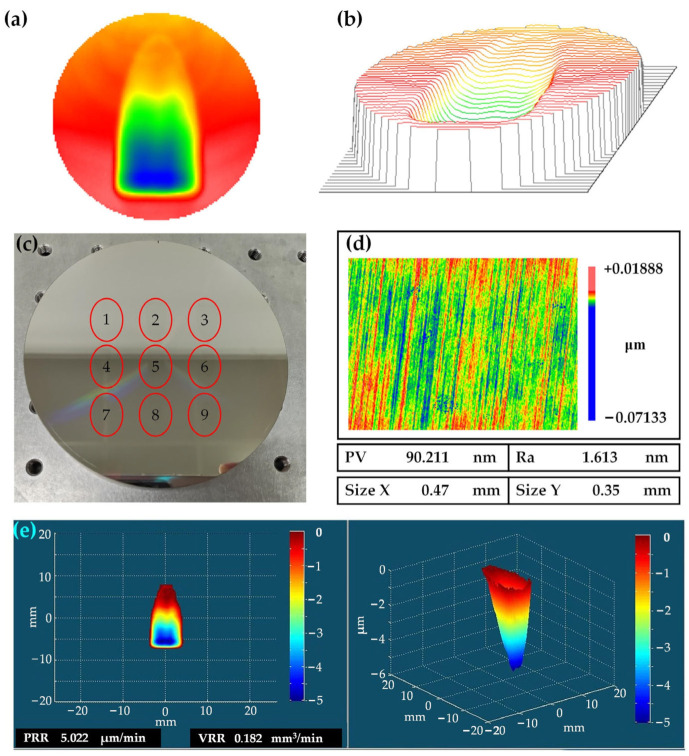
Removal function: (**a**) two-dimensional plot of Removal Function No. 2; (**b**) three-dimensional plot of Removal Function No. 2; (**c**) nine polishing points of removal function on the workpiece; (**d**) the roughness of Polishing Point No. 2; (**e**) PRR and VRR of Polishing Point No. 2 μm^3^.

Nine removal function tests were carried out to produce MRF removal function polishing points, as shown in Table 3. The polishing points were created by first using the initial surface shape of the NiP-coated workpiece as measured by a laser interferometer (Zygo Verifire, Zygo Corporation, Connecticut, USA). Then, nine polishing points were produced at different locations on the workpiece surface using an MRF machine according to the nine sets of parameters in Table 3. Finally, the surface shape of the machined NiP-coated workpiece was examined using the laser interferometer, and the surface shape of the workpiece before and after MRF machining was subtracted to obtain the removal function [35]. A two-dimensional view of the test Polishing Point No. 2 is shown in Figure 7a, and a three-dimensional view of the test Polishing Point No. 2 is shown in Figure 7b. The nine polishing points prepared are shown in Figure 7c. The roughness of each polishing point was measured using a white-light interferometer (Zygo NewView 700S, Zygo Corporation, Connecticut, USA) and is shown in Figure 7d for Polishing Point No. 2. The PRR and VRR were calculated in the self-developed process software. Figure 7e shows the calculation results for Polishing Point No. 2. The roughness, PRR, and VRR are listed for each group of polishing points in Table 2.

## 5. Results and Discussion

### 5.1. Weight Analysis and Level Optimization of Factors

After completing the tests and collecting the data, the relevant data were subjected to extreme variance analysis. The analysis of extreme variance balances the effect of other factors on the results when considering a single factor so that the differences caused by each level are caused by the factor itself [36]. The results of the extreme variance analysis are shown in Table 4.

**Figure 8:**
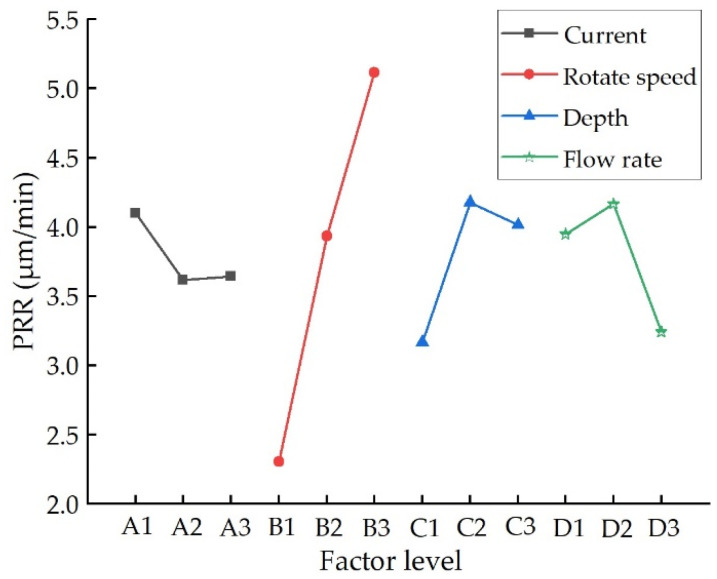
The relationship between PRR and process parameters.

A factor–indicator relationship diagram is usually applied to represent the results of orthogonal experiments. The level of each factor is taken as the horizontal coordinate, and the mean value of the indicator is taken as the vertical coordinate. Figure 8 shows the relationship between PRR and process parameters. It can be seen that in the MRF processing of NiP coating, the influence of each process parameter on PRR is in the following order: polishing wheel rotation speed > press-in depth of ribbon > fluid flow rate > magnetic field strength. The effect of polishing wheel rotation speed on PRR is the most significant. The main reason is that as the speed of the polishing wheel increases, the relative velocity between the magnetorheological fluid ribbon and the surface of the workpiece increases. According to Equation (8), the PRR has a linear relationship with the relative velocity ideally, and an increase in the speed of the polishing wheel will cause an increase in the pressure field in the polishing area, thus significantly increasing the PRR of the material.

**Figure 9:**
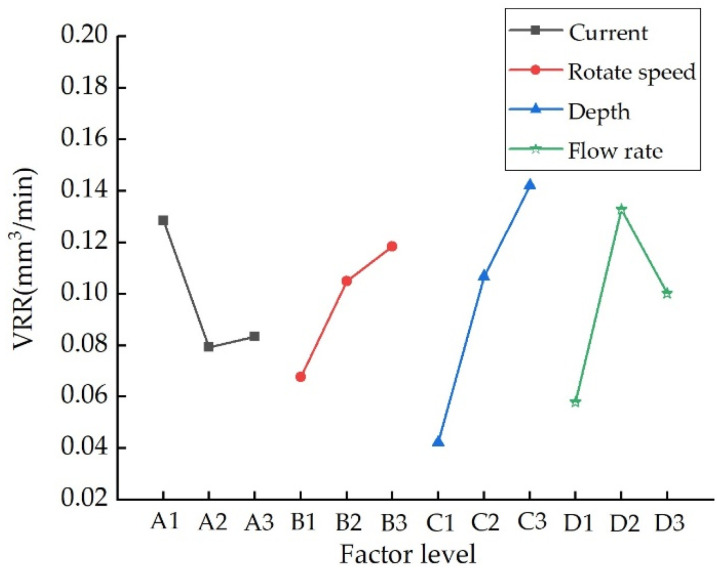
The relationship between VRR and process parameters.

Figure 9 shows the relationship between VRR and process parameters. It can be seen that in the MRF processing of NiP coating, the influence of each process parameter on VRR is in the following order: press-in depth of ribbon > fluid flow rate > polishing wheel rotation speed > magnetic field strength. The most significant effect on the VRR is that of the press-in depth of ribbon. The main reason for this is that as the depth of indentation increases, the geometry of the removal function increases significantly. Therefore, the VRR increases substantially. An increase in rotational speed also increases the VRR. Although the geometry of the removal function shrinks as the polishing wheel rotation speed increases, the VRR also increases as the PRR increases significantly.

**Figure 10:**
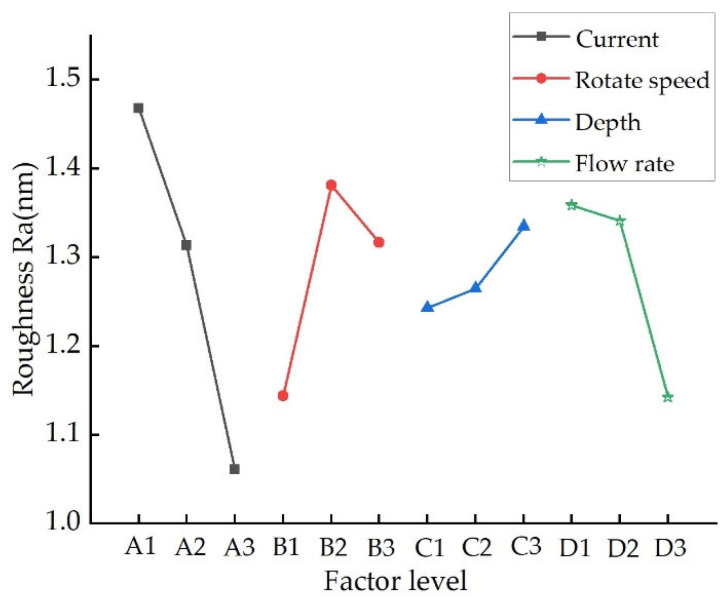
The relationship between roughness and process parameters.

Figure 10 shows the relationship between the surface roughness and the process parameters. It can be seen that in the MRF processing of NiP coating, the influence of each process parameter on the surface roughness is in the following order: magnetic field strength > polishing wheel rotation speed > fluid flow rate > press-in depth of ribbon. The effect of magnetic field strength on surface roughness is the most significant. The main reason for this is that as the magnetic field strength increases, the shear yield strength of the magnetorheological fluid increases. As a result, the hardness of the flexible polishing die formed by the magnetorheological fluid ribbon and its holding power on the abrasive grains will increase, thus increasing the effective indentation depth and effective indentation area of the abrasive grains. As the magnetic field strength increases, the pressure in the polished area increases, which increases the effective indentation depth of the abrasive grains and the effective indentation area.

Based on the above analysis, it can be seen that when machining NiP coatings through MRF, we can select suitable machining process parameters according to the initial conditions of the workpiece. In the case of large material removal, for example, when the turning tool patterns of the NiP coating need to be removed quickly or when the surface shape has a poor error, we can choose a process parameter with a high material removal rate, A1B3C2D2 or A1B3C3D2 in Table 4. In the case of low material removal and high surface quality requirements for the NiP coating, the process parameter can be chosen that gives excellent surface roughness, A3B1C1D3 in Table 4.

### 5.2. Processing Results and Discussion of NiP Coating through MRF

**Figure 11:**
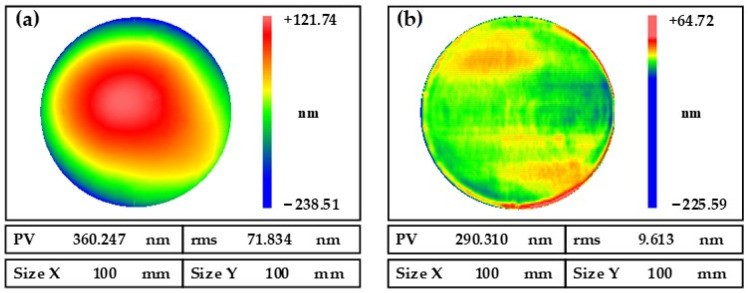
Comparison of surface shape errors: (**a**) before MRF; (**b**) after MRF.

The NiP coating workpiece was obtained by electroless nickel plating on an aluminum alloy substrate with a 100 mm diameter. The NiP coating was first subjected to SPDT turning to give the surface a quick mirror finish. Figure 11 shows the surface shape of a NiP coating workpiece inspected using a laser interferometer (Zygo Verifire, Zygo Corporation, Connecticut, USA). As shown in Figure 11a, the surface shape error of the NiP coating after SPDT turning is RMS 71.834 nm. An MRF fluid with silicon carbide as abrasive grains was specially developed for the NiP coating processing, and the MRF rapid reshaping of the NiP coating was carried out using the A1B3C3D2 process parameters in Table 3 No. 2. As shown in Figure 11b, after 16 min of processing, the surface shape error of the NiP coating converged to RMS 9.613 nm, with a convergence ratio of 7.5.

**Figure 12:**
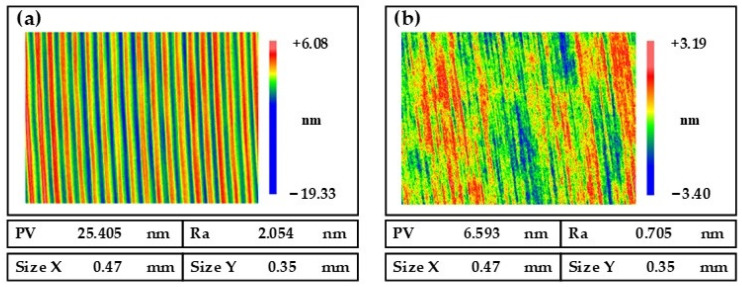
Comparison of surface roughness: (**a**) before MRF; (**b**) after MRF.

Figure 12 shows the surface roughness of the NiP coating measured by a white-light interferometer (Zygo NewView 700S) under a 20 × lens with a scan size of 0.47 mm × 0.35 mm. Figure 12a shows the SPDT machined NiP coating with a measured surface roughness of Ra 2.054 nm. Then, the NiP coating was processed by MRF, as shown in Figure 12b, and the surface roughness improved to Ra 0.705 nm.

### 5.3. Discussion on Surface Performance of NiP Coating

**Figure 13:**
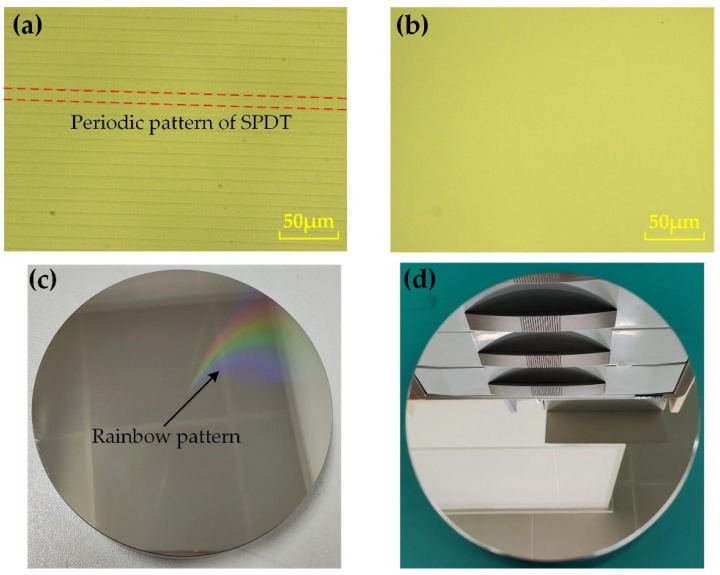
NiP coating: (**a**) microstructure before MRF; (**b**) microstructure after MRF; (**c**) workpieces processed through SPDT; (**d**) workpieces processed through MRF.

Figure 13a,b shows the microstructure of the NiP coating as observed by the digital microscope (Keyence VHX-600E, Osaka, Japan). Figure 13a shows the microstructure of the NiP coating machined by SPDT turning. Regular periodic turning patterns on the surface of the workpiece can be observed, which increase scattering and reduce the imaging quality of the optical system [20]. Hence, a subsequent process is required to remove the turning pattern [12]. Figure 13b shows the microstructure of the NiP coating machined by MRF, and it can be observed that the periodic turning pattern on the surface is completely removed, and the finished surface of the workpiece is free from machining defects such as scratches, pits, and cracks. Figure 13c shows a physical NiP coating workpiece machined by SPDT turning. We could find rainbow patterns on the surface of the workpiece, which are caused by the periodic tool patterns. Figure 13d shows a NiP coating workpiece machined by MRF. It can be seen that the rainbow pattern on the surface of the workpiece has completely disappeared because MRF machining has removed the cyclical tool pattern generated by the SPDT machining of the NiP coating workpiece.

**Figure 14:**
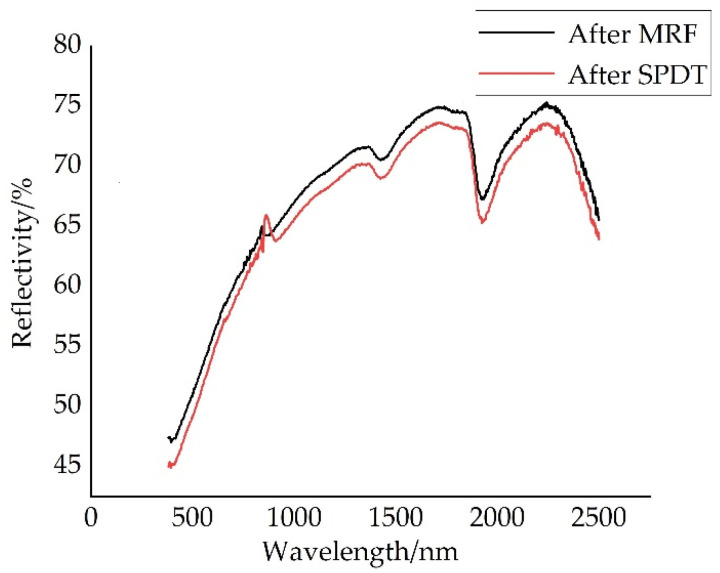
Reflectivity of NiP coating.

Figure 14 shows the results of the NiP coating reflectance test using a spectrophotometer (HITACHI U4100, Tokyo, Japan). The range of spectral bands tested is 380 nm to 2500 nm, including the visible and infrared bands [39]. It can be seen from Figure 14 that the reflectivity of NiP coating processed by MRF is enhanced in both the visible and infrared wavelengths.

In Ref. [21], SPDT turning tool marks were removed by smoothing polishing on a NiP coating flat workpiece of Φ100 mm. The surface roughness of the NiP coating reached Ra 0.7 nm, which took about 1 h. In Ref. [22], SPDT turning tool marks were removed by float polishing on a NiP-coated flat workpiece of Φ50 mm. The surface roughness of the NiP coating reached Ra 0.7 nm, which took about 2 h. In contrast, in Section 5.2, MRF took only 16 min. Therefore, compared to other polishing methods for NiP coating, the outstanding advantage of MRF is that it can maintain a high machining efficiency while obtaining an ultra-smooth machined surface.

## 6. Conclusions

With its unique material removal mechanism, stable removal function, extremely high face convergence efficiency, and surface quality super-smooth capability, MRF technology has a broad development prospect. MRF technology is an excellent tool for processing NiP coatings. It is possible to quickly correct surface shape errors and process coatings with an ultra-smooth surface using MRF technology to process NiP coatings. MRF technology has become one of the effective machining methods for the efficient and automated manufacture of high-precision optical parts.

In this paper, we investigated the method of MRF technology for processing NiP coating to achieve higher precision and realize higher application requirements. We developed an MRF fluid dedicated to the MRF polishing of NiP coatings and optimized the machining process of the MRF polishing of NiP coatings through experiments. Compared with other polishing methods, MRF processing of NiP coatings can maintain high processing efficiency while obtaining ultra-smooth machined surfaces. As a deterministic machining technology, MRF can rely on precise inspection and machining to achieve quantitative removal of workpiece face shape errors, which has unique advantages in the ultra-precision manufacturing of NiP coatings.

## Figures and Tables

**Table 1:** Range of parameters for MRF.

Parameters	Current (A)	Wheel Speed (rpm)	Pressed Depth (mm)	Flow Rate (mL/s)
Numerical	6.5~7.5	150~210	0.2~0.4	80~120

**Table 2:** Level-factor table for the orthogonal test.

Factor	A Current (A)	B Wheel Speed (rpm)	C Pressed Depth (mm)	D Flow Rate (mL/s)
1	6.5	150	0.2	80
2	7	180	0.3	100
3	7.5	210	0.4	120

**Table 3:** Experimental results of orthogonal process parameters.

No.	Current (A)	Wheel Speed (rpm)	Pressed Depth (mm)	Flow Rate (mL/s)	PRR (μm/min)	VRR (mm^3^/min)	Roughness Ra (nm)
1	6.5	150	0.2	80	2.163	0.00515	1.371
2	6.5	180	0.3	100	5.022	0.182	1.613
3	6.5	210	0.4	120	5.116	0.198	1.420
4	7	150	0.3	120	1.982	0.0628	1.022
5	7	180	0.4	80	4.158	0.0933	1.546
6	7	210	0.2	100	4.707	0.0816	1.372
7	7.5	150	0.4	100	4.772	0.135	1.039
8	7.5	180	0.2	120	2.630	0.0398	0.985
9	7.5	210	0.3	80	5.528	0.0754	1.159

**Table 4:** Range analysis of a single factor.

Object	Factors	Ranges	Order	Preferred Combination
PRR	A	0.484	B > C > D > A	A1B3C2D2
	B	2.811		
	C	1.01		
	D	0.927		
VRR	A	0.04915	C > D > B > A	A1B3C3D2
	B	0.05068		
	C	0.09992		
	D	0.07492		
Roughness	A	0.407	A > B > D > C	A3B1C1D3
	B	0.237		
	C	0.092		
	D	0.217		

## Data Availability

Not applicable.
